# Electrochemical Characterization and Simulation of Ion Transport in Anion Exchange Membranes for Water Treatment Applications

**DOI:** 10.3390/membranes15040123

**Published:** 2025-04-13

**Authors:** Qiaolin Lang, Yang Liu, Gaojuan Guo, Yang Zhang

**Affiliations:** 1 Shandong Engineering Research Centre for Pollution Control and Resource Valorization in Chemical Industry, College of Environment and Safety Engineering, Qingdao University of Science and Technology, Qingdao 266042, China; langql@qibebt.ac.cn (Q.L.); liuyang@qust.edu.cn (Y.L.); 2 Qingdao Institute of Bioenergy and Bioprocess Technology, Chinese Academy of Sciences, Qingdao 266101, China; 3 National Center for Materials Service Safety, University of Science and Technology Beijing, Beijing 100083, China

**Keywords:** ion exchange membrane, electrochemical characterization, chronopotentiometry, current–voltage curve, electrochemical impedance spectroscopy, COMSOL model

## Abstract

This study presents a comprehensive electrochemical characterization and simulation of anion exchange membranes (AEMs) for water treatment applications, focusing on ion transport behavior. Experimental techniques, including chronopotentiometry, current–voltage (I–V) curve measurements, and electrochemical impedance spectroscopy (EIS), were employed to investigate the kinetics and dynamics of ion transport at the membrane interface. The results were validated and further explored through finite element method (FEM) simulations using COMSOL Multiphysics. The study revealed key insights into the role of membrane resistance, ion diffusion, and capacitive effects on overall membrane performance. Parametric analyses of electrolyte layer thickness, bulk solution concentration, and membrane porosity provided guidelines for optimizing membrane design. The findings highlight the importance of considering these factors in enhancing the efficiency and applicability of AEMs in water treatment processes. Future work will focus on refining simulation models and exploring advanced materials to further improve membrane performance.

## 1. Introduction

With the increasing stringency of wastewater discharge standards, water treatment has become an essential technology for ensuring environmental safety. The ion exchange membranes (IEMs) has emerged as a crucial part in various water treatment technologies, including electrodialysis, diffusion dialysis, and membrane capacitive deionization [1,2,3,4,5]. These membranes are designed to selectively transport anions or cations, thereby facilitating the separation and purification of ionic species from aqueous solutions. The performance of IEMs is highly dependent on their intrinsic properties, such as ion exchange capacity, membrane resistance, and porosity, as well as the interactions between the membrane and the surrounding electrolyte solution [6,7,8,9]. Therefore, a comprehensive understanding of the ion transport behavior within these membranes is essential for optimizing their design and enhancing their efficiency in water treatment applications [10].

Over the past few decades, significant advancements have been made in the development [11,12,13] and characterization of ion exchange membranes [8]. Techniques such as scanning electron microscopy (SEM), atomic force microscopy (AFM), ion exchange capacity measurements, and contact angle measurements have been employed to characterize the physical and chemical properties of ion exchange membranes [14,15,16]. These studies have revealed that the transport behavior of ions within the membrane and flow channels plays a decisive role in the recovery of target solutions and energy efficiency.

In particular, electrochemical characterization methods have gained significant attention due to their ability to provide detailed information about the kinetics and dynamics of ion transport at the membrane interface [17,18]. Chronopotentiometry [19,20], current–voltage (I–V) curve measurements [21], and electrochemical impedance spectroscopy (EIS) [22,23] are among the most widely used techniques in the electrochemical characterization of IEM. Chronopotentiometry involves applying a constant current to the membrane and measuring the resulting potential change over time, thereby offering insights into the kinetics of ion transport and electrochemical reactions. The I–V curve measurement characterizes the electrochemical behavior of the membrane, including its ionic conductivity and resistance under different operating conditions. EIS, on the other hand, is a non-destructive technique that provides information about the membrane’s ionic transport properties and resistance by analyzing the impedance response over a wide range of frequencies.

Recent studies have demonstrated that the electrical double layer (EDL) formed at the membrane–electrolyte interface significantly influences ion transport behavior [23]. The structure and properties of the EDL can be affected by various factors, including the membrane’s surface charge density, the concentration of the electrolyte solution, and the presence of supporting electrolytes [24]. Understanding the role of the EDL in ion transport is crucial for optimizing membrane performance and minimizing energy consumption in water treatment processes [25,26,27].

The existing electrochemical characterization techniques typically operate at the microscopic scale, providing valuable insights into the overall behavior of ion exchange membranes. However, they are limited in their ability to directly observe the interactions between ions and the membrane at the nanometer level. To overcome the limitations of traditional electrochemical characterization techniques, simulation methods [28], particularly those based on the finite element method (FEM), offer significant advantages for studying ion transport at the nanometer level [29,30]. By using powerful software platforms like COMSOL Multiphysics 6.1, researchers can create highly detailed models that incorporate the complex geometries and physical phenomena occurring at the nanoscale [30]. This enables the investigation of ion interactions with the membrane surface, the influence of surface charge density, and the effects of membrane porosity on ion transport, all of which are critical for understanding and optimizing membrane performance [31,32]. Additionally, simulations can be easily adjusted to explore different scenarios and parameters, providing a flexible and cost-effective way to study ion transport mechanisms and predict the behavior of ion exchange membranes under various conditions [33].

In this study, we performed the systematic electrochemical characterization and simulation of anion exchange membranes (AEMs) to gain a deeper understanding of ion transport behavior. Commercial AEMs were tested using chronopotentiometry, I–V curve measurements, and EIS. The experimental results were validated through finite element method (FEM) simulations using COMSOL Multiphysics^®^ [34], a powerful software platform for creating models and simulation applications. Two models were established to simulate chronopotentiometry, I–V curve measurements, and EIS, respectively. This study aimed to provide a comprehensive analysis of the ion transport mechanisms through AEMs and offer valuable insights for optimizing membrane design and application in separation processes, ultimately contributing to the development of more efficient and sustainable water treatment technologies.

## 2. Experimental Methods

### 2.1. Materials and Reagents

Commercial anion exchange membranes were purchased from Shandong Tianwei Membrane Technology Co., Ltd. (Weifang, China). The technical specifications for the commercial anion exchange membrane are listed in Table 1. The main reagent used in the electrochemical characterization experiments was sodium chloride (≥99%, Sinopharm Chemical Reagent Co., Ltd. Shanghai, China). The supporting electrolyte solution was 100 mM NaCl.

### 2.2. Apparatus

**Figure 1:**
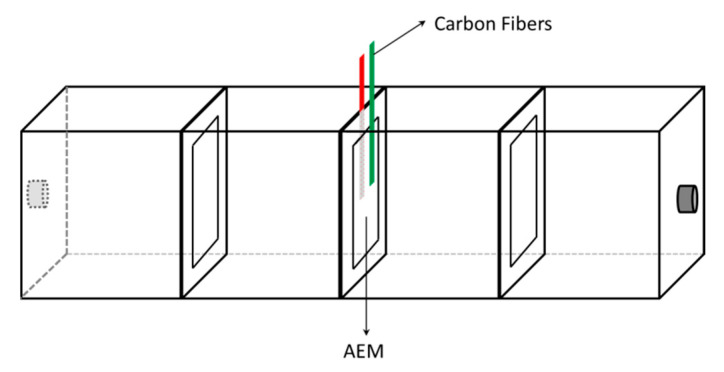
Scheme of four-chamber electrodialysis cell used in the electrochemical characterization of AEM.

The electrochemical characterization of AEMs was performed using a CHI760E electrochemical workstation (Shanghai CH Instruments Inc., Shanghai, China). And a four-chamber electrodialysis cell was employed as the holder of the AEMs (As shown in Figure 1). The compartments contiguous with the AEMs were filled with the supporting electrolyte solution. During the experiments, the solution in the compartments was not circulated to eliminate convective mass transfer effects. The volume of each chamber in the membrane stack was 8 cm^3^. And the effective contact area of the membrane was 2 cm × 2 cm. Carbon fibers were used as electrodes fixed in contact with both sides of the AEM. During the electrochemical characterization tests, the current or voltage was applied via two carbon fiber electrodes. Specifically, one end of the carbon fiber was connected to the working electrode alligator clip, while the other end was connected to both the reference and counter electrode alligator clips.

### 2.3. Electrochemical Characterization Methods

Chronopotentiometry is a powerful technique for studying the kinetics of ion transport and electrochemical reactions at the membrane interface. The test involves applying a constant current to the membrane and measuring the resulting potential change over time. The test was conducted in two segments: the first segment involved a resting period with zero current applied, while the second segment applied a constant current density of 1.0 mA/cm^2^ for 48 s.

The current–voltage (I–V) curve measurement aims to characterize the electrochemical behavior of the ion exchange membrane, including its ionic conductivity and resistance under different operating conditions. The measurement was performed using linear sweep voltammetry (LSV) on the electrochemical workstation. The potential was swept linearly from 0 V to 2 V at a scan rate of 0.1 V/s, and the corresponding current response was recorded to obtain the I–V curve.

EIS is a non-destructive technique for characterizing ion exchange membranes. It provides information about the membrane’s ionic transport properties and resistance. The EIS measurements were conducted by applying a sinusoidal voltage with an amplitude of 5 mV using the electrochemical workstation. The frequency range was set from 1 Hz to 10^5^ Hz. The impedance data were analyzed using Nyquist and Bode plots to extract information about the membrane’s resistance, capacitance, and ion transport behavior.

## 3. Simulation Methods

The COMSOL Multiphysics^®^ is a software platform for creating physics-based models and simulation applications. Two models were created in this work to simulate the chronopotentiometry, I–V curve, and EIS tests on the AEMs.

### 3.1. Modeling Details

#### 3.1.1. Setting Details for Chronopotentiometry and I–V Curve Modeling

**Figure 2:**
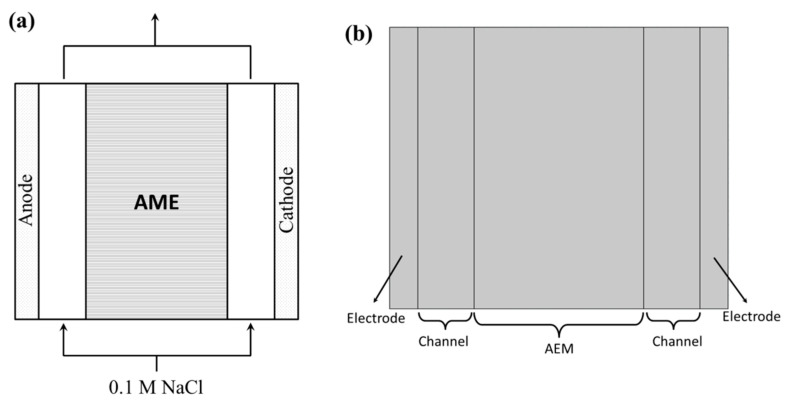
(**a**) The configuration of membrane stacks for AEM electrochemical characterization experiment; (**b**) the geometry of modeling in chronopotentiometry and I–V curve simulation.

A two-dimensional model was chosen for the chronopotentiometry and I–V curve simulation. The parameters were set for the test conditions required in subsequent studies, that is, the applied current density *i*_app_ for chronopotentiometry modeling and the voltage *V*_1_ for I–V curve simulation. Based on the schematic diagram of the electrochemical characterization device for the membrane, its geometric schematic diagram is shown in Figure 2a. The membrane to be tested was fixed in the center position, with porous electrodes (carbon fiber electrodes in actual experiments) on both sides. The electrodes and the membrane were separated by flow channels. Since the carbon fiber electrodes were not in close contact with the membrane surface in the actual experiment, the geometric construction of the flow channel was set to be a small size to simulate this situation. In the electrochemical characterization of the membrane, the solutions on both sides of membrane were connected outside. Thus, in the geometric model, the solution in the flow channel was set to 0.1 M NaCl on both sides, with the lower boundary as the inlet and the upper boundary as the outlet. The height of the membrane stack and the width of the membrane, two flow channels, and two carbon fiber electrodes were 100 μm and 60, 20 and 10 μm, respectively (Figure 2b).

The main material involved in the model was the diluted electrolyte solution, with the value of the electrical conductivity of 5.5 × 10^−6^ S·m^−1^ and electrolyte conductivity of 2.0 S·m^−1^. The parameter of applied current density and voltage were also set for the parametric scanning studies, with the initial value of 1.0 mA·cm^−2^ and 0.5 V, respectively.

#### 3.1.2. Setting Details for EIS Modeling

Considering the frequency domain perturbation was applied linearly to the system, a one dimensional model was chosen for EIS simulation. The parameters about the geometry and frequency used in the EIS simulation are listed in Table 2. The value of the double-layer capacitance *Cdl* was set to 10 μF/cm^2^, referring to the general value for oxide electrodes. The initial electrolyte concentration *c_bulk* was set to 0.1 M, and the membrane length *L_m* was set to 60 μm. The impedance test frequency range was set from 1 Hz to 10,000 Hz. The exchange current density and the number of electrons transferred for the cathode was specified. Then, parameters for the anode were set to enforce the electroneutrality condition of the system. The value of the exchange current density was higher than the general reference values due to the high roughness of the carbon fiber electrode, which can enhance the exchange current density [35]. And the value of exchange current density for the cathode was usually much higher than that of the anode [36].

**Figure 3:**
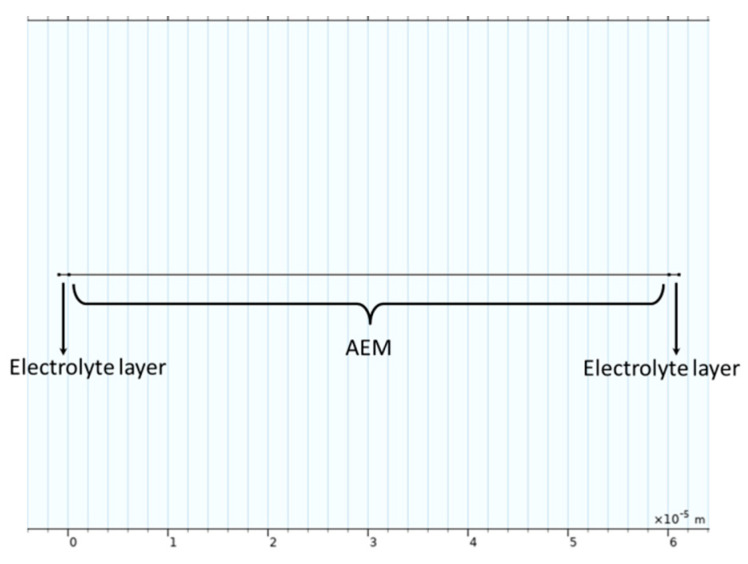
Geometry of EIS simulation of membrane.

The geometric scheme is shown in Figure 3. Corresponding to the actual experimental operation, no external force stirring was applied during the impedance test. And since the impedance disturbance was an alternate current (AC), the electrodes on both sides of the membrane did not need to be distinguished as positive or negative. The impedance simulation results of the membrane were also verified through experimental data.

### 3.2. Governing Equations

For the modeling of chronopotentiometry and I–V curve, the governing equations were the same. The electrochemical characterization process of the membrane involves three types of ion transport in the electrolyte, that is, diffusion, electro-migration, and convection. The transfer of ions in the electrolyte can be described by the Nernst–Planck equation,

(1) $${\vec{J}}_{i}=-D_{i}\nabla c_{i}-z_{i}u_{i}c_{i}F\nabla \phi_{l}+c_{i}\vec{u}$$

where ${\vec{J}}_{i}$ is the flux of ion *i*; $D_{i}$, $c_{i}$, $z_{i}$ and $u_{i}$ are the diffusion coefficient, concentration, charge number, and mobility of ion *i*, respectively; and *F* and $\phi_{l}$ are the Faraday constant and electric potential. The first, second, and third terms in the equation represent the flux of ion *i* caused by diffusion, electro-migration, and convection, respectively.

The local current density $i_{\mathrm{ct}}$ at the electrode surface can be expressed by the Butler–Volmer equation,

(2) $$i_{\mathrm{ct}}=i_{0}\left\{C_{R}\left(\frac{\alpha_{a}F\eta }{RT}\right)-C_{O}\exp\left(\frac{-\alpha_{c}F\eta }{RT}\right)\right\}$$

where $i_{0}$ is the exchange current density; $C_{R}$ and $C_{O}$ are the dimensionless concentrations of the reducing and oxidizing substances, respectively; $\alpha_{a}$ and $\alpha_{c}$ are the anodic and cathodic transfer coefficients, respectively; and *η*, *R*, and ***T*** are the overpotential, ideal gas constant, and temperature of the electrolyte in Kelvin. The first and second terms in the equation represent the contributions of the anodic and cathodic currents under applied potential, respectively.

For EIS simulation, the governing equation retained the diffusion and convection terms but neglected the migration term compared with the Nernst–Planck equation of chronopotentiometry and I–V modeling. The reason was that in the EIS simulation, it was assumed that there was a large quantity of supporting electrolyte, and thus, the resistance of the solution was sufficiently low and the electric field could be negligible [34]. The governing equation then became

(3) $${\vec{J}}_{i}=-D_{i}\nabla c_{i}+c_{i}\vec{u}$$

And the equation of current density at electrodes’ surface was also from the Butler–Volmer equation, which was the same as Equation (2).

### 3.3. Boundary Conditions

#### 3.3.1. Boundary Conditions for Chronopotentiometry and I–V Curve Modeling

The lower boundary of the flow channel was the inlet, and the upper boundary was the outlet. In addition, there was no mass transfer of substances at other external boundaries of the model. The current in the model only involved the direction along the x-axis, so the upper and lower boundaries of the model were set as insulating boundaries.

The diffusion coefficient of the substance in AEM was set to an ideal situation, where the diffusion coefficient of cations was zero and the diffusion coefficient of anions was consistent with the value of the anions in the electrolyte.

The electrodes used in experiment were carbon fibers, and thus, the electrodes were modeled as porous electrodes. Since the electrodes involved Faradaic reactions, relevant settings for porous electrode reactions were added. In this simulation, the electrolyte was a dilute NaCl solution, so the Faradaic electrode reactions involved were only water electrolysis reactions. To achieve the electrochemical polarization curve test, the outermost boundaries of the model were set as electrolyte potentials. The left side was set as electrolyte potential 1, with an initial value of 0 V, and the right side was set as electrolyte potential 2, with an initial value set as the parameter *V*_1_ to be scanned, which was used for the subsequent polarization curve test.

#### 3.3.2. Boundary Conditions for EIS Modeling

The left end of the line segment was set as a no-flux boundary, and the right end was set as an insulator. The initial concentration was set as parameter *c_bulk* in the parameter list for purpose of subsequent parametric scanning studies. The initial potential was set to 0 V, which meant the impedance scan was carried out under the condition of no external potential applied. The impedance test is based on the double-layer theory at the interface. Therefore, at a certain distance, it is necessary to set the ion concentration equal to the bulk solution concentration to provide the concentration boundary condition for the double layer. On the right side of this model, it was set as a concentration boundary condition, where the concentrations of substances were equal to the bulk solution concentration *c_bulk* at this point.

The left end point was set as electrode surface and included electrode reaction settings. The electrode reaction kinetics expression was also based on the Butler–Volmer equation. A double-layer capacitance was set to specify the size of the double-layer capacitance under the AC signal of impedance, with the value of Cdl in the parameter list for subsequent parametric scanning studies. And the middle segment area was set as membrane while the parts between membrane and electrodes were set as electrolyte layers.

## 4. Results and Discussion

### 4.1. Chronopotentiometry Tests and Simulation Results

**Figure 4:**
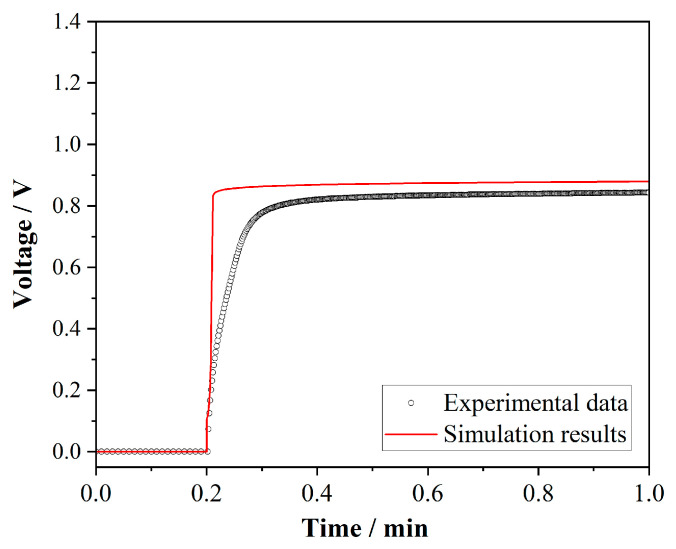
Chronopotentiometry curve of the commercial anion exchange membrane. Applied current density of 1.0 mA/cm^2^.

The chronopotentiometry results for the commercial anion exchange membrane are shown in Figure 4 (dots). The potential difference across the membrane increased rapidly when the constant current was applied. The initial rapid increase in potential was attributed to the depletion of ions at the membrane surface, leading to a concentration polarization effect. As time progressed, the potential increased slowly and eventually reached a steady state. The transition time (τ) from the initial rapid increase to the steady state was determined to be approximately 4.4 s. This result was consistent with the theoretical calculation based on Sand’s equation, which predicts a transition time of 3.17 s under the experimental conditions [37,38]. Moreover, the transition time was also on the same scale as that reported by E. Volodina et al. [39].

The simulation results were compared with the experimental data, as shown in Figure 4. It can be seen from the comparison that the voltage drop in the 0–0.2 min segment was calculated as a difference, so both the experimental and simulation results were 0, and the experimental and simulation results fit well in this segment. Similarly to the experimental results, the simulation results also did not show the first stage in the chronopotentiometric curve. The transition time τ in the second segment of the experimental results was longer, while the voltage change in the simulation results was faster, with the transition time of 1.2 s. The reason was that the system in the simulation was smaller, while the actual experimental membrane area and the volume of the electrolyte solution were larger. Therefore, under the same applied current density, the concentration polarization in the simulation system was faster, and the transition time was shorter. Another possible reason was that the ions underwent adsorption on the electrode surface caused by the porous structure of the carbon fiber, which was not accounted for in the modeling settings. While in the second segment (0.2–1.0 min), both chronopotentiometric curves leveled off, indicating that the system reached a stable state. The experimental data and simulation results converged, with the voltage drop around 0.8 V. This conformance suggested that the simulation model primarily presented the steady-state behavior of the AEM. And the only difference existed in the transition period, which was attributed to the simplifications inherent in the simulation settings.

### 4.2. Current–Voltage (I–V) Tests and Simulation Results

**Figure 5:**
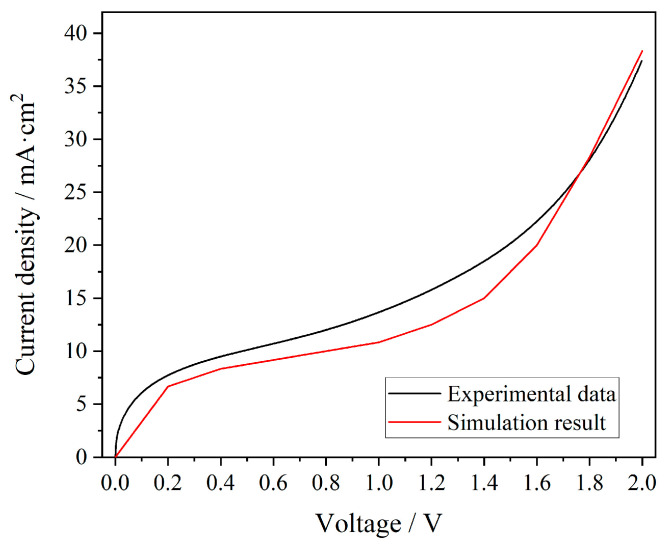
Current–voltage curve of the commercial anion exchange membrane.

The current–voltage (I–V) curve test of the membrane was carried out using the two-electrode linear sweep voltammetry (LSV) method, and the results are shown in Figure 5 (black line). The voltage test range was 0–2 V, and the corresponding current values have been converted into current density units. In the first stage, the ohmic region, as the applied voltage starts from 0 V, ions migrated through the ion exchange membrane under the Influence of the electric field, and the migration of ions caused a rapid increase in current. In the second stage, the limiting current region, the number of ions passing through the membrane had reached its maximum value, and the system started a stable stage. Even with the continuous increase in voltage, the increase in current was relatively gentle. Thus, the intersection of the tangents of the first and second stages corresponded to the limiting current density value *j*_lim_ of the membrane. In the third stage, the over-limit current region, when the voltage reached around 1.2 V, due to the concentration polarization effect, the ion concentration at the membrane surface was depleted, and the ions in the bulk solution could not be replenished in time. Therefore, the water dissociation reaction started on the membrane surface [40], generating a large number of hydrogen ions and hydroxide ions, which became new charge carriers, thereby causing a rapid increase in the current density. With the increase in voltage, the rate of water splitting increased, and the trend of a current increase also accelerated, presenting the slope of the I–V curve increasing rapidly. There have been many reports on the I–V curve test of ion exchange membranes, and the results are also three-stage curves, with explanations given for the limiting current density [21,41,42,43,44,45,46]. When the concentration polarization in the system is eliminated by other interfering factors, the current platform stage due to concentration polarization will not appear in the system. Therefore, in the I–V curve of the membrane, the current increases linearly with the increase in voltage [47].

The simulation results were basically consistent with the experimental data. In the practical test, the current density in the first segment of the I–V curve increased faster, indicating that there were more ions that could act as charge carriers in the actual test system. In the second segment, the current platform was more obvious in the simulation results. The reason was still that the contact area of the membrane in the actual test system was larger, and the diffusion flux of ions passing through the membrane continued to increase with the increase in voltage. Therefore, the increase in charge carriers led to a continuous increase in current density. The third stage involved the process of water splitting, and the experimental and simulation results fit well.

### 4.3. Electrochemical Impedance Spectroscopy (EIS) Tests and Simulation Results

**Figure 6:**
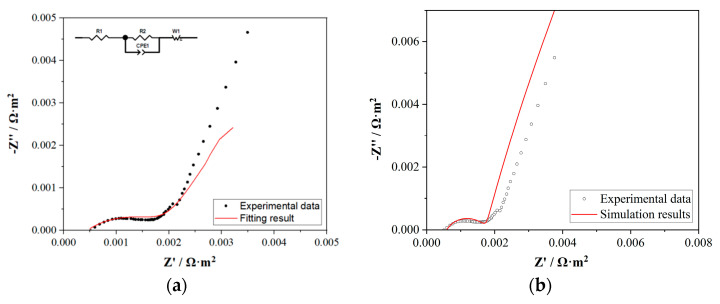
Electrochemical impedance spectroscopy results: (**a**) experimental data and fitting results of the equivalent circuit, (**b**) experimental data and simulation results.

The impedance test results of the ion exchange membrane in 100 mM NaCl are shown in Figure 6a (dots). In the high-frequency region, the intersection of the Nyquist curve with the x-axis represented the ohmic resistance in the system. In this study, this value was the sum of the ionic resistance across the membrane *R*_m_ and the electronic transfer resistance at the electrode surface *R*_e_, with a value of 5.1 × 10^−4^ Ω·m^2^ (5.1 Ω·cm^2^). The reference value of this commercial ion exchange membrane was 4.5–5.5 Ω·cm^2^ (Table 1), indicating the good conformance between the experimental data and the value given by AEM’s manufactory.

The impedance test data were studied with an equivalent circuit, and the fitting results are shown in Figure 6a (red line). The inset in Figure 6a shows the equivalent circuit results. The equivalent circuit presented a well-matched line with the experimental data. The resistor R1 in the equivalent circuit represents the ohmic resistance in the high-frequency region (*R*_m_ + *R*_e_), in which *R*_m_ was the resistance of membrane and *R*_e_ was the ohmic resistance of the electrolyte. In the medium-frequency region, the capacitive arc was not a standard semicircle, so the fitting circuit result was a constant phase element (CPE) CPE1 in parallel with a resistor R2. CPE1 represented the capacitive behavior of charge transfer within the membrane, and R2 represented the resistance of charge transfer at the membrane surface. In electrochemical systems, an ideal capacitive element (such as parallel plate capacitor) in the equivalent circuit usually required uniform charge distribution and complete smooth electrode surfaces. However, in actual electrochemical interfaces, the electrode surface was roughness, inhomogeneous, or had non-uniform charge distribution, which could cause the capacitive behavior to deviate from the ideal state. In the impedance test of the membrane, due to the non-smooth membrane surface, the roughness caused uneven charge storage within the membrane, which was equivalent to a CPE rather than a capacitor element in the equivalent circuit fitting. The low-frequency region of the diffusion behavior fitting result was the Warburg impedance W1, representing the diffusion behavior of charges from the double-layer surface of the membrane to the bulk solution, with the slope representing the value of the diffusion resistance. The bigger slope of the experimental data than the fitting result indicated a larger diffusion resistance in the actual system.

The components of the equivalent circuit were basically consistent with those reported in the literature for the impedance spectra of AEMs, with only slight differences in the low-frequency diffusion region [48,49,50]. The reason was that the frequency domain below 1 Hz was not tested in this experiment. Since the focus was on the interaction between charge and the membrane surface, the behavior of diffusion in lower frequency domain was not the primary research interest in this work. The parameters used in the fitting of equivalent circuit are listed in Table 3, in which the unit of resistance given by the fitting software was ohm, while the unit in the experimental and simulation results was converted to the unit of membrane surface resistance (Ω·cm^2^). The membrane surface resistance is a parameter conventionally used in the electrochemical characterization of ion exchange membranes. The fitting resistance values in ohms were converted to membrane surface resistivity. The resulting high-frequency resistance and charge transfer resistance at the membrane surface were 4.476 Ω·cm^2^ and 10.76 Ω·cm^2^, respectively, which are basically consistent with the readings from the experimental impedance spectra.

The simulation results of the AC impedance study are shown in Figure 6b (red line), in which the Nyquist plots showed good agreement with the experimental results. The high-frequency region represented the resistance of *R*_m_ and *R*_e_, with a value of 5.39 × 10^−4^ Ω·m^2^ (5.39 Ω·cm^2^), which was very close to the reference membrane resistance given by the manufactory (4.5–5.5 Ω·cm^2^). In the mid-frequency region, the shape, height, and span of the capacitive arc were essentially consistent with the experimental results, indicating that the simulation of charge transfer behavior at the membrane surface was validated. In the low-frequency diffusion region, the simulated result was an ideal Warburg impedance diffusion line, while the experimental result showed some disturbances in this area and did not form ideal Warburg diffusion impedance. These phenomena were possibly caused by the membrane or electrode surface heterogeneity [51] or the change in electrolyte under the applied voltage.

### 4.4. Effect of Electrolyte Properties on EIS Simulation Results

#### 4.4.1. Effect of Electrolyte Layer Thickness

**Figure 7:**
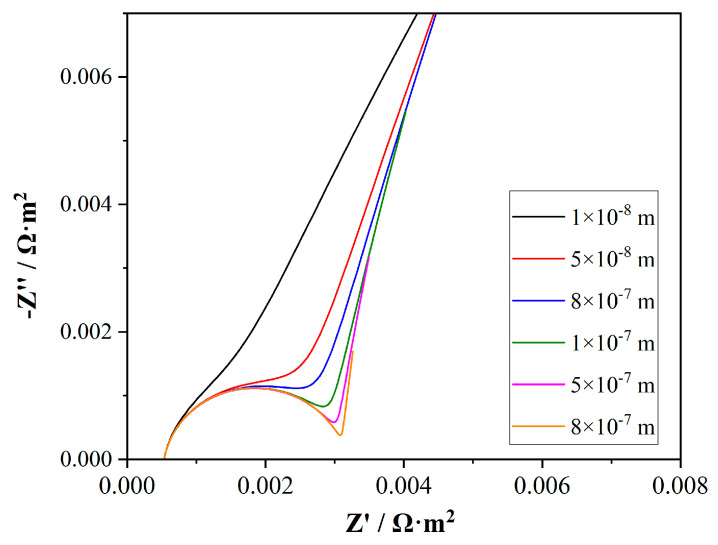
Simulation results of Nyquist plot from parametric sweep research on thickness of electrolyte layer.

A parametric sweep was conducted on the electrolyte layer thickness parameter *L_s*, ranging from 1 × 10^−8^ m to 8 × 10^−7^ m. As shown in Figure 7, the impedance spectra revealed a significant dependence of the charge transfer resistance on the electrode–membrane distance. The main differences in the Nyquist plots for different electrolyte layer thicknesses were in the mid-frequency region, which was due to the behavior differences in the ion transfer process at the membrane surface caused by the distance between the membrane surface and the electrons. As the distance increased, the diameter of the semi-circle also increased, indicating a higher charge transfer resistance. The reason was attributed to the increased difficulty in electron transfer across a larger distance, which results in a higher resistance. The smallest semi-circle, corresponding to the smallest distance of 1 × 10^−8^ m, suggested the lowest charge transfer resistance. It was expected as the proximity of the electrode to the membrane surface facilitated easier charge transfer, thereby reducing the resistance. When the thickness of the electrolyte layer between the membrane and the electrode reached 8 × 10^−7^ m, the resistance to charge transfer increased, forming an essentially complete capacitive arc. In the experiments, the capacitive arc was not obvious, possibly because the gap between the electrode and the membrane was relatively small, and due to the actual membrane and electrode surfaces were both relatively rough, resulting in uneven electrolyte layer.

Combining the above simulation results with the actual experimental test, the Nyquist plot of the AEM shows curves closer to 1 × 10^−7^ m and 5 × 10^−8^ m, which indicated that the distance in real test was about 1 × 10^−7^ m. Through the parametric sweep research, for the actual Nyquist results of the membrane, certain membrane surface information could be provided based on the shape of the capacitive arc.

#### 4.4.2. Effect of Bulk Solution Concentration

**Figure 8:**
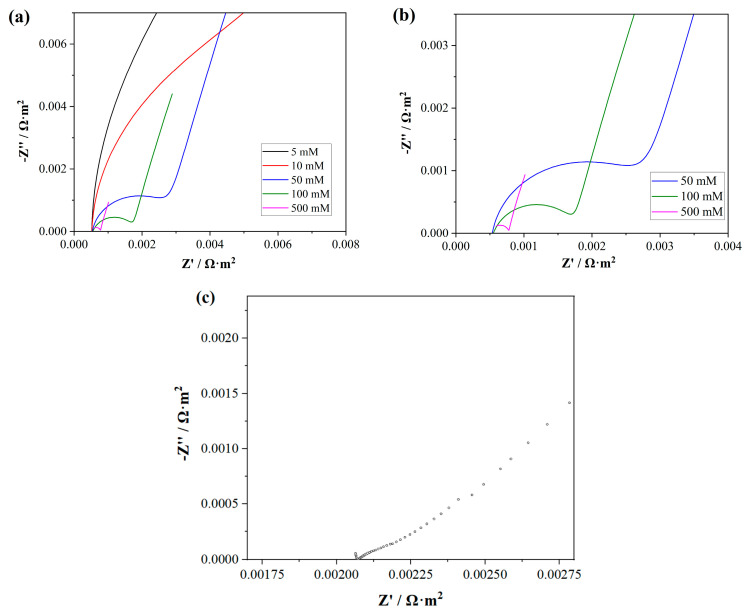
Simulation results of Nyquist plot from parametric sweep research of bulk concentration: (**a**) bulk concentration from 5 to 500 mM; (**b**) enlarged view for the concentration of 50–500 mM; (**c**) Nyquist plot from experimental test in 500 mM NaCl solution.

The concentration of the electrolyte solution was also a key factor affecting the charge transfer properties through membrane. Therefore, a parametric sweep study was conducted on the bulk solution concentration parameter *c_bulk*, with a range set from 5 to 500 mol/m^3^ (5–500 mM). It was obvious from Figure 8a that as the electrolyte concentration increases, the resistance values of both the real and imaginary parts of the impedance decreased significantly. The reason was that with the increase in electrolyte concentration, the concentration of ions increased, which enhanced the conductivity of the solution. This improvement in conductivity facilitated the movement of charge carriers, thereby reducing the charge transfer resistance.

A detailed enlargement of the range from 50 to 500 mM is shown in Figure 8b. When the bulk solution concentration was 500 mM, the capacitive arc was not shown in the high-frequency region, indicating that the electrolyte concentration was high enough for the electrolyte layer to act as a conductor. This simulation result at 500 mM was observed when the AEM was tested in the same concentration of NaCl solution in the experiment (Figure 8c).

### 4.5. Effect of Membrane Electrolyte Volume Fraction

In multi-physics simulation, the electrolyte volume fraction *ε* is an important parameter for porous substrates (various types of membranes and porous electrodes). It refers to the proportion of the volume occupied by the electrolyte in porous electrodes or media. It reflects the distribution of the electrolyte in the porous structure and is commonly used to describe the effective volume of the electrolyte in porous separator. The electrolyte volume fraction affects the transport paths of ions and their diffusion rates, thereby influencing the electrochemical performance of porous substrates.

**Figure 9:**
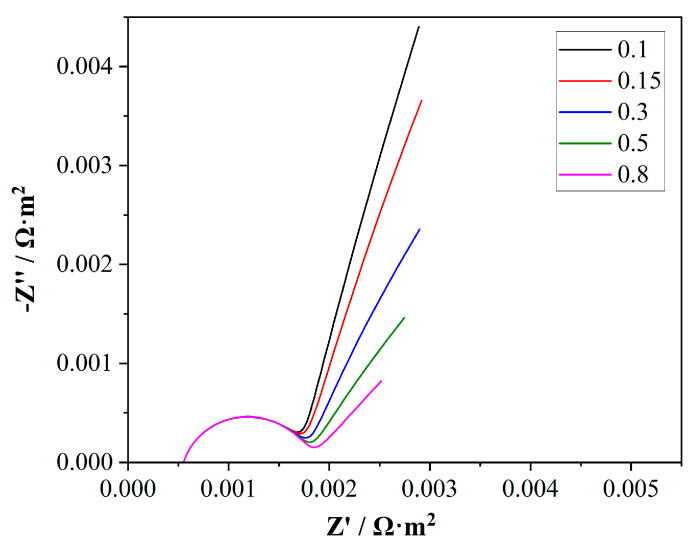
Simulation results of Nyquist plot from parametric sweep research on electrolyte volume fraction *ε*.

In this study, a parametric sweep was conducted on the electrolyte volume fraction of the membrane, ranging from 0.1 to 0.8, with the results shown in Figure 9. From the Nyquist results, it was observed that under the settings of this model, the electrolyte volume fraction of the membrane had a relatively small effect on the capacitive arc in the mid-frequency region. As the *ε* increased, the capacitive arc slightly tended to become more complete. However, the more noticeable change occurred in the slope of the Warburg impedance in the diffusion region. With the enlargement of the *ε* value, the charge diffusion impedance decreased, thereby reducing the slope of the Warburg impedance.

## 5. Conclusions

This study provides a comprehensive investigation of ion transport behavior in anion exchange membranes (AEMs) for water treatment applications through a combination of electrochemical characterization and finite element method (FEM) simulations. The results obtained from chronopotentiometry, current–voltage (I–V) curve measurements, and electrochemical impedance spectroscopy (EIS) were validated and further explored using COMSOL Multiphysics simulations, offering valuable insights into the fundamental mechanisms governing ion transport within these membranes. The transition time (τ) in chronopotentiometry tests from the initial rapid increase to a steady state was experimentally determined to be approximately 4.4 s, consistent with theoretical predictions based on Sand’s equation. The simulation results closely matched the experimental data, indicating the rapid reaching of state status for the AEM under constant current. The I–V curve measurements demonstrated a characteristic three-stage behavior: an initial ohmic region with rapid current increase, a limiting current region with a plateau, and an over-limit current region marked by a sharp rise in current density due to water dissociation. The simulation results were in good agreement with the experimental data, indicating that the current density in the first segment increased more rapidly in the actual test system due to the availability of more charge carriers. The EIS analysis provided detailed information on the membrane’s resistance, capacitance, and ion transport behavior across different frequency ranges. The experimental Nyquist plots were successfully fitted with an equivalent circuit model, revealing the contributions of ohmic resistance, charge transfer resistance, and diffusion resistance to the overall impedance response. The simulation results closely matched the experimental data, with the high-frequency region indicating a membrane resistance of 5.39 × 10^−4^ Ω·m^2^ (5.39 Ω·cm^2^). The parametric sweep studies on electrolyte layer thickness, bulk solution concentration, and membrane electrolyte volume fraction provided critical insights into the factors influencing ion transport and membrane performance. The results indicated that the thickness of the electrolyte and the electrolyte volume fraction within the membrane were key parameters affecting the capacitive and diffusion behaviors. The electrochemical characterization experiments and simulations facilitated the understanding of the ions transporting mechanisms through the membrane.

## Figures and Tables

**Table 1:** Technical specifications of the commercial anion exchange membrane.

Technical Specifications	Units	Value
Ion Exchange Capacity	mmol·g^−1^	0.90–1.10
Thickness (Wet)	μm	60–80
Water Content (25 °C)	wt %	20–25
Membrane Resistance	Ω·cm^2^	4.5–5.5
Translation Number	-	≥0.98
Stability	pH	1–14

**Table 2:** List of parameters in the model of EIS characterization of AEM.

Parameters	Value	Description
*Da*	1 × 10^−9^ m^2^/s	Diffusion coefficient of anion
*Dc*	1 × 10^−11^ m^2^/s	Diffusion coefficient of cation
*c_bulk*	100 mol/m^3^	Bulk concentration
*Cdl*	0.1 F/m^2^	Double-layer interfacial capacitance
*i0ref*	964.85 A/m^2^	Reference exchange current density
*freq_min*	1 Hz	Minimum frequency
*freq_max*	10,000 Hz	Maximum frequency
*L_m*	6 × 10^−5^ m	Membrane length
*A_el*	1 × 10^−4^ m^2^	Electrode area
*V_app*	0.005 V	Applied perturbation potential
*ε*	0.15	Electrolyte volume fraction in membrane
*L_s*	1 × 10^−7^ m	Electrolyte layer (space between membrane and electrode) length

**Table 3:** Equivalent circuit fitting parameters.

Element	Value	Error (%)
R1 (Ω)	4.476	0.10
R2 (Ω)	10.76	10.33
CPE1-T (F)	0.00019018	4.91
CPE1-P	0.49158	5.47
W1-R (Ω)	10.91	9.17
W1-T (F)	0.0064176	19.87
W1-P	0.40078	19.84

## Data Availability

The data that support the findings of this study are available from the corresponding author upon reasonable request.
